# Effects of Composite LED Light on Root Growth and Antioxidant Capacity of *Cunninghamia lanceolata* Tissue Culture Seedlings

**DOI:** 10.1038/s41598-019-46139-2

**Published:** 2019-07-05

**Authors:** Yuanyuan Xu, Yuyao Liang, Mei Yang

**Affiliations:** 1 Guangxi Key Laboratory of Forest Ecology and Conservation, College of Forestry, Guangxi University, Nanning, 530004 Guangxi PR China; 2 College of Forestry, Beijing Forestry University, Beijing, 100083 PR China; 3 College of resources and environment, Fujian Agriculture and Forestry University, Fuzhou, 350002 Fujian PR China

**Keywords:** Forestry, Plant breeding

## Abstract

We used the 12^th^ generation of the *Cunninghamia (C.) lanceolata* tissue culture seedlings, and white light emitting diode (LED) light as control (CK). We applied five composite LED light treatments, red-blue 4:1, 8:1 (4R1B and 8R1B), red-blue-purple 8:1:1 (8R1B1P), and red-blue-purple-green 6:1:1:1, 8:1:1:1 (6R1B1P1G and 8R1B1P1G), to study the effects of light quality on root growth characteristics and antioxidant capacity of *C. lanceolata* tissue culture seedlings. The results showed that: (1) rooting rate, average root number, root length, root surface area, and root activity were higher with 6R1B1P1G and 8R1B1P1G treatments compared to 4R1B, 8R1B, 8R1B1P and CK treatments; and the root growth parameters under the 8R1B1P1G treatment were as high as 95.50% for rooting rate, 4.63 per plant of the average number of root, 5.95 cm root length, 1.92 cm^2^ surface area, and 145.56 ng/(g·h) root activity, respectively. (2) The composite lights of 4R1B, 8R1B, 8R1B1P, 6R1B1P1G, and 8R1B1P1G are beneficial for the accumulation of soluble sugar content (SSC) and soluble protein content (SPC), but not conducive for the increase of free proline content (FPC); the plants under 6R1B1P1G and 8R1B1P1G treatments had higher superoxide dismutase (SOD), peroxidase (POD), catalase (CAT), ascorbate peroxidase (APX) activity and lower malondialdehyde (MDA), polyphenol oxidase (PPO) activity. (3) Redundancy analysis showed that POD activity positively correlated with root activity; SPC, SOD and CAT activities positively correlated with root growth parameters; while SSC, MDA content, APX and PPO activities negatively correlated with root growth parameters. These results suggest that the responses of root growth and antioxidant capacity of the *C. lanceolata* tissue culture seedlings to different light qualities vary. The relationship between root growth parameters and antioxidant capacity was closely related. Red-blue-purple-green was the most suitable composite light quality for root growth of *C. lanceolata* tissue culture seedlings, and 8:1:1:1 was the optimal ratio, under which the rooting rate, root activity and root growth of tissue culture seedlings peaked.

## Introduction

Light quality, i.e., the spectrum of different wavelengths, is an important environmental factor affecting the growth, development, morphogenesis, and physiological metabolism of plants^1^. The photosynthetically active radiation required for plant growth has a wavelength of 400–700 nm, of which red and orange lights (wavelength between 610–720 nm) and blue and purple lights (wavelength between 400–510 nm) account for approximately 85%, and 12%, respectively, while yellow and green lights (wavelength between 510–610 nm) were minimally absorbed^2^. The light sources generally used for greenhouse are fluorescent, metal halide, high-pressure sodium, and incandescent lamps. However, these sources contain unnecessary wavelengths that are of low quality for promoting growth^3^. By comparison, light-emitting diode (LED) can directly convert electrical energy into light energy, which has the advantages including high luminous efficiency, low energy consumption, cold light source, small volume, long life, environment protection, energy saving and easy regulation. It is an ideal illuminant for plant facility cultivation to regulate the light environment and has broad application prospects in plant tissue culture^4–8^.

The absorption of the light spectrum is specific to plants. Red light plays an important role in controlling chloroplast, stem, petiole growth and reproductive system function, while blue light mainly regulates plant growth, leaf expansion, photomorphogenesis, stomatal opening, photosynthesis and pigment accumulation^9^. And the spectra of red and blue lights are similar to those required for plant photosynthesis, so most studies focus on evaluating the effects of monochrome or mixed red and blue LED. The effects of white, far red, green, yellow and orange LEDs have been reported only in a few studies. Previous studies have shown that monochromatic light can promote root growth and physiological metabolism, and it varies with plant species. For example, red LED blocks root growth of *Doritaenopsis* plants^10^, while blue LED promotes root formation of *Chrysanthemum* plants^11^ and increases the rooting rate and root number of *Achillea millefolium* culture^12^ and *Vanilla planifolia* plantlets^13^. In physiological aspect, red light can improve the antioxidant activity of pea seedlings^14^, *Dendrobium officinale* seedlings^15^, and *Eleutherococcus senticosus* somatic embryos^16^, and the response of different enzymes to different light quality is different. In addition, Liu *et al*.^17^ also pointed out that red light was beneficial to the accumulation of carbohydrates in *Oncidium* protocorm-like bodies (PLBs), while blue light was beneficial to the improvement of protein content and antioxidant enzyme activities.

When it comes to red and blue combination light, Li *et al*.^18^ showed that the root activity, sucrose, starch and SSC of *Gossypium hirsutum* tissue culture seedlings under red light were the highest, while red LED with a large number of blue light could make the plants larger, healthier and biomass larger. Shin *et al*.^10^ pointed out that the combination of red and blue light promoted the root growth and the increase of leaf area of *Doritaenopsis* tissue culture seedlings, and the biosynthesis of carbohydrates (starch, sucrose, glucose and fructose) under this light quality was significantly higher than that under monochrome red light, blue light and fluorescent light treatment. In addition, red-blue light can also increase the content of protein in *Vigna radiata*^19^, and metabolites such as beta-anthocyanin in red leaf cultivars of *Atriplex hortensis*^20^. Generally speaking, the response of different plants to the combination of red and blue light is different, and the effects of different ratio of red and blue light on plants are also different. Naznin *et al*.^9^ indicated that 83% R + 17% B treatment was beneficial to the antioxidant capacity of lettuce (*Lactuca sativa*), spinach (*Spinacea oleracea*) and cabbage (*Brassica oleracea* var. *sabellica*), while 91% R + 9% B treatment was beneficial to the antioxidant capacity of *Ocimum basilicum* and *Capsicum annuum*. Furthermore, different cultivars of the same plant have different adaptability to different light environments^21,22^.

Less research has been done on the root growth and physiology of plant under other combinations light. Kim *et al*.^23^ found that adding green fluorescent lamps to red-blue LEDs could promote the growth of lettuce and produce more biomass than that under cold white fluorescent lamps. Ren *et al*.^24^ showed that the root length and root activity of *Phalaenopsis* were improved with red/blue/far red LED 3:6:1. Lin *et al*.^25^ pointed out that the combination of red, blue and white light was beneficial to the increase of SSC in lettuce (*Lactuca sativa* L. var. *capitata*) compared with the combination of red and blue light. It could be seen that the biological effect of monochrome light may not be inferior to that of red-blue composite light or other composite light, and the ideal light environment for each plant is unique. The light quality and light intensity that affect the growth and development of one plant may not produce similar results for another plant.

With the development of modern forestry, clonal forestry guided by modern genetics and breeding theory has gradually come into our vision. It mainly uses the most suitable plants for management purposes as clonal original plants on the basis of selection, and produces clonal afforestation seedlings through large-scale multiplication^26^. Plant tissue culture provides a favorable way for clonal seedling breeding. LED has been widely used in plant tissue culture, vegetable production and seedling cultivation in plant factories, but most of them are concentrated in herbaceous species. The application of LED in woody plants is still rare. Previous research showed that a combination of red and blue light produced the highest percentage of shoot regeneration of *Populus euramericana*^27^ and significantly increased blueberry (*Vaccinium corymbosum* L.) shoot and root biomass^28^.

*Cunninghamia lanceolata* (Lamb.) Hook. (Taxodiaceae, *Cunninghamia*), a native tree species in southern China, is a dominant tree species in southern China because of its adaptability to diverse environmental conditions, and is widely planted commercially for its rapid growth and the properties of its highly durable, scented wood. It is currently grown on over 11 million hectares, accounting for 20–30% of the total commercial timber production in China^29,30^. At present, the research on tissue culture seedlings rooting of *C. lanceolata* mostly focused on the adjustment of basic medium and growth regulators^31,32^. Few studies were performed on microenvironments such as light conditions. Ding *et al*.^33^ showed that monochrome red LED and monochrome blue LED were used as light source for tissue culture of *C. lanceolata* in turn, which could promote its rooting and seedling growth. Zhou *et al*.^34^ pointed out that monochromatic red, blue, green LED or composite light of the three light were beneficial for the increase of chlorophyll content and fluorescence parameters of *C. lanceolata*. However, studies for the application of two or more LED composite lights on the rooting stage tissue culture of *C. lanceolata* were not reported. Gupta *et al*.^35,36^ indicated that there was a close relationship between the changes of antioxidant enzymes induced by LED and shoot organogenesis. Furthermore, the interaction between antioxidant enzymes and promoters played an important role in plant regeneration *in vitro*. What we are interested in is whether there is a relationship between the root organogenesis and the antioxidant capacity of *C. lanceolata* plants in tissue culture. What are the conditions of LED suitable for rooting stage culture in tissue culture of *C. lanceolata*? Therefore, the main purpose of this experiment was to study the effects of different light quality components and ratios of LED on root growth and antioxidant capacity of *C. lanceolata* tissue culture seedlings, to explore the relationship between root growth and antioxidant capacity, and to screen out the most favorable light quality components and ratios. This study would provide reference for the regulation of light quality environment of *C. lanceolata* superior clone and other woody plants.

## Results

### Effects of different light qualities on root growth characteristics of *C. lanceolata* tissue culture seedlings

**Figure 1:**
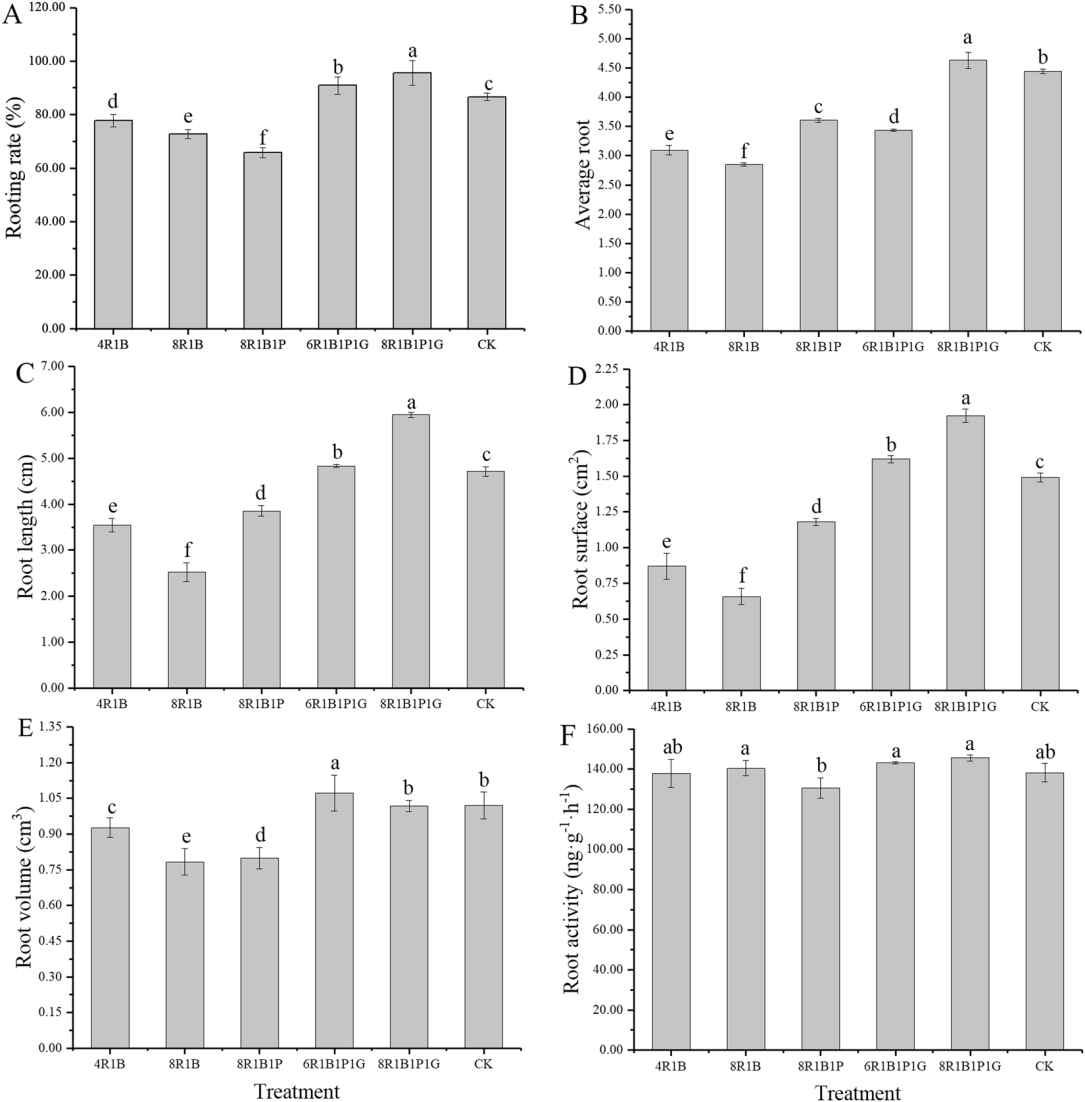
Effect of different light quality on root growth of *C. lanceolata* seedlings. Note: (**A**) Rooting rate; (**B**) Average root; (**C**) Root length; (**D**) Root surface; (**E**) Root volume; (**F**) Root activity. The root parameters were determined at LED light quality 4R1B, 8R1B, 8R1B1P, 6R1B1P1G, 8R1B1P1G, and CK. All samples were taken at the end of the light periods. Data are means of three experiments ± standard deviation. Different letters (a, b, c, d, e) indicate significant difference between treatments at P ≤ 0.05 by using Duncan’s multiple range test.

**Figure 2:**
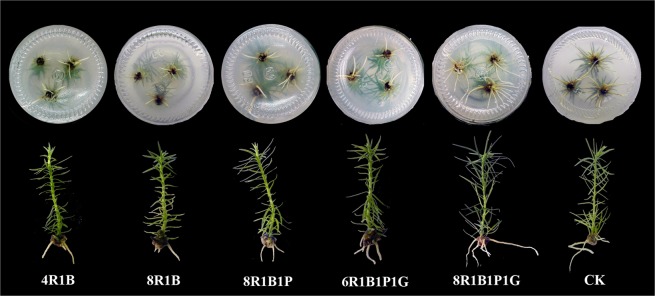
Growth characteristics of *C. lanceolata* seedlings under different light quality.

Rooting rate is the percentage of the number of rooted seedlings divided by the total number of remaining seedlings. Root activity refers to the activity ability of physiological functions of plant roots, and it is determined by the strength of its respiratory function^37^. As shown in Fig. 1, different light qualities had significant effects on the rooting rate, average root number, root length, root surface area, root volume and root activity of *C. lanceolata* tissue culture seedlings (P < 0.05). Under the 8R1B1P1G treatment a maximum of 95.50% rooting rate, 4.63 per seedling average root number, 5.95 cm length, 1.92 cm^2^ surface area and 145.56 ng/(g·h) root activity was measured, which was 10.28%, 4.20%, 26.23%, 28.91%, and 5.33%, respectively, higher than those in the CK treatment group. Meanwhile, the desired seedling was obtained under the 8R1B1P1G light treatment, the root and leaf were many and long, and distributed evenly (Fig. 2). The root volume reached its maximum under the 6R1B1P1G treatment. The rooting rate, average root number, root length, root surface area, and root volume were significantly smaller in the 4R1B, 8R1B and 8R1B1P treatment group compared to the CK treatment group. In general, root growth and root activity were superior under the red-blue-purple-green composite light compared to red-blue and red-blue-purple.

### Effects of different light qualities on the SSC, SPC and FPC in the leaves of *C. lanceolata* tissue culture seedlings

The SSC, SPC and FPC of the *C. lanceolata* tissue culture seedlings under different light qualities were significantly different (P < 0.05) (Table 1). The SSC under different composite light was higher than that under the CK treatment. The difference between 4R1B, 8R1B, and 8R1B1P and CK was significant. The SPC under 4R1B, 8R1B1P, 6R1B1P1G and 8R1B1P1G were significantly higher compared to CK. In addition, the SPC under red-blue-purple and red-blue-purple-green light quality was higher than that under red-blue. The FPC under the 4R1B and 8R1B1P1G, and CK treatments were comparable, while it were significantly lower in the 8R1B, 8R1B1P and 6R1B1P1G treatment compared to CK. In general, the SPC and FPC under the 4R1B treatment were significantly higher than 8R1B, while the SPC and FPC with 6R1B1P1G treatment were significantly lower compared to 8R1B1P1G.

**Table 1:** Contents of SSC, SPC and FPC in leaves of *C. lanceolata* seedlings under different light qualities.

Light treatment	SSC/ (mg·g^−1^)	SPC/ (mg·g^−1^)	FPC/ (ug·g^−1^)
4R1B	20.14 ± 0.69 a	84.17 ± 0.49 d	5.53 ± 0.08 a
8R1B	19.36 ± 0.20 abc	78.95 ± 1.17 e	5.38 ± 0.08 d
8R1B1P	19.62 ± 0.59 ab	108.45 ± 1.45 a	5.42 ± 0.17 c
6R1B1P1G	19.02 ± 0.56 bcd	87.59 ± 0.57 c	5.45 ± 0.12 b
8R1B1P1G	18.59 ± 0.70 cd	90.85 ± 2.48 b	5.54 ± 0.06 a
CK	18.32 ± 0.16 d	79.87 ± 0.732 e	5.54 ± 0.24 a

Note: All samples were taken at the end of the light periods. Data are means of three experiments ± standard deviation. Different letters (a, b, c, d, e) in the same column indicate significant difference between treatments at P ≤ 0.05 by using Duncan’s multiple range test.

### Effects of different light qualities on antioxidant capacity in the leaves of *C. lanceolata* tissue culture seedlings

Table 2 shows that different light qualities have significant effects on the MDA content and antioxidant enzyme activities in the leaves of *C. lanceolata* tissue culture seedlings (P < 0.05). The MDA content in the 6R1B1P1G and 8R1B1P1G treatment groups were significantly lower compared to CK and the other composite light, while SOD, POD and CAT were significantly higher than others. And the POD and CAT activities were highest under the 8R1B1P1G treatment. The APX activity was significantly higher in the 8R1B and the 8R1B1P1G treatment groups compared to CK treatment, while the APX activity under the 4R1B, 8R1B1P, and 6R1B1P1G treatments did not differ much from that of the CK treatment. The PPO activity was as follows: 8R1B1P>8R1B>CK>6R1B1P1G>8R1B1P1G>4R1B.

**Table 2:** MDA content and antioxidant enzyme activities in leaves of *C. lanceolata* seedlings under different light qualities.

Light treatment	MDA/ (n mol·g^−1^)	SOD/ (U·g^−−1^)	POD/ (U·min^−1^·g^−1^)	CAT/ (U·min^−1^·g^−1^)	APX/ (U·min^−1^·g^−1^)	PPO/ (U·min^−1^·g^−1^)
4R1B	1.24 ± 0.04 a	141.87 ± 4.02 f	400.80 ± 7.63 e	49.33 ± 3.71 c	146.93 ± 6.42 c	533.24 ± 5.13 c
8R1B	1.27 ± 0.03 a	165.06 ± 6.31 e	479.20 ± 6.94 d	36.71 ± 4.03 d	240.40 ± 4.33 a	595.73 ± 11.29 a
8R1B1P	1.21 ± 0.12 ab	337.59 ± 6.22 c	587.47 ± 11.87 c	63.47 ± 6.00 b	148.80 ± 5.00 c	604.16 ± 8.43 a
6R1B1P1G	0.93 ± 0.05 c	366.77 ± 9.70 a	725.60 ± 5.00 b	57.53 ± 3.90 b	142.81 ± 1.73 c	544.44 ± 3.87 c
8R1B1P1G	0.80 ± 0.01 d	352.67 ± 2.69 b	771.20 ± 9.73 a	102.20 ± 3.70 a	174.67 ± 8.33 b	537.24 ± 7.93 c
CK	1.16 ± 0.03 b	179.98 ± 8.69 d	196.27 ± 4.11 f	45.87 ± 3.10 c	144.27 ± 6.55 c	566.22 ± 5.04 b

Note: All samples were taken at the end of the light periods. Data are means of three experiments ± standard deviation. Different letters (a, b, c, d, e, f) in the same column indicate significant difference between treatments at P ≤ 0.05 by using Duncan’s multiple range test.

### Relationship between root growth and antioxidant capacity

**Figure 3:**
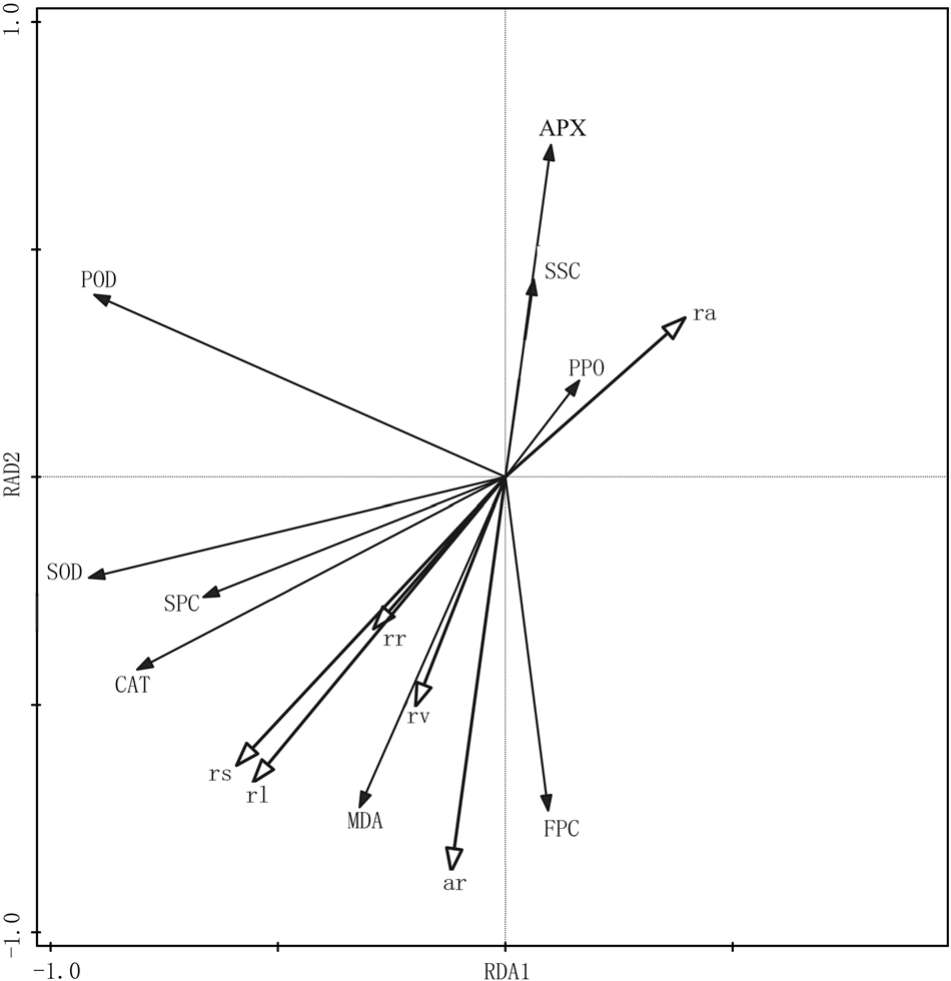
Redundancy analysis of the relationship between root growth characteristics and antioxidant capacity characteristics and antioxidant capacity. rr, ar, rl, rs, rv, ra, SSC, FPC, SPC, MDA, SOD, POD, CAT, APX, PPO stand for rooting rate, average root, root length, root surface area, root volume, root activity, soluble sugar content, free proline content, soluble protein content, malondialdehyde content, superoxide dismutase activity, peroxidase activity, catalase activity, ascorbate peroxidase activity, polyphenol oxidase activity, respectively.

The RDA sorting diagram of redundant analysis reflects the relationship between root growth and antioxidant capacity (Fig. 3). The hollow arrow in the figure indicates the root growth parameters, and the solid arrow indicates the antioxidant capacity parameters. Table 3 depicts the correlation coefficient between the first two axes of RDA sorting diagram of the root growth parameters and antioxidant capacity parameters, in which the cumulative explanatory variable of the first and the second axes is 88.88%, indicating that the first two axes reflect most of the information in the relationship between root growth and antioxidant capacity. In the RDA sorting diagram, the length of the solid arrow line projection on the sorting axis indicates the correlation between the antioxidant capacity parameters and the sorting axis. The first axis of Fig. 3 contains most of the antioxidant capacity parameters information. The length of the solid arrow connection indicates the correlation between the root growth parameters and antioxidant capacity parameters variation. The longer the connection line, the greater is the correlation. In Fig. 3, the connections of the FPC, SPC, MDA, and the activities of SOD, POD, CAT, and APX are longer, indicating that these parameters have a greater influence on root growth. The angle between the hollow and solid arrow line represented the correlation between a root growth parameter and a antioxidant capacity parameters, which is numerically equal to the cosine of its angle. In Fig. 3, the POD activity positively correlated with root activity and SPC. SOD, CAT activity positively correlated with rooting rate and surface area and root length; while SSC, MDA content, APX, PPO activity negatively correlated with rooting rate, root length, surface area, volume, and average root number.

**Table 3:** Correlation coefficients of first two RDA axes of root growth parameters and antioxidant capacity parameters

Parameters	First axis	Second axis
SSC	0.0749	0.5281
FPC	0.1031	−0.6863
SPC	−0.6582	−0.2492
MDA	0.7499	0.2772
SOD	−0.9173	−0.2719
POD	−0.9129	0.3944
CAT	−0.8158	−0.3840
APX	0.0900	0.7867
PPO	0.1650	0.2136
Explained fitted variation (cumulative)	73.22%	88.88%

## Discussion

The effects of light quality on root growth of tissue culture seedlings varied with plant species^38–40^. In our study, different light qualities had significant effects on the root development of the *C. lanceolata* tissue culture seedlings. In general, the root growth and root activity was higher under the red-blue-purple-green composite light compared to the red-blue and red-blue-purple composite lights. The root growth parameter values under the red-blue-purple-green composite light were higher than those under the CK treatment, while the root growth under the red-blue and red-blue-purple composite lights was reduced compared to CK treatment. The tissue culture seedlings had higher rooting rate, length, surface area, volume, and root activity under the red-blue-purple-green composite light, which is due to larger root absorption area and vigor of plants, that will increase the absorption capacity of the roots and promote growth. This effect is based on the red-blue-purple composite light that produced a distinct complementary effect. Purple and green lights are important for photosynthesis radiation^41^, although the demand for plants for this light composition is small. The supplementation of purple and green light, in addition to the main red and blue lights, may promote the distribution of photosynthates to roots and then promote the growth of roots. Moreover, the rooting rate, average root number, length, surface area, and root activity reached a maximum under the 8R1B1P1G treatment, indicating that red-blue-purple-green composite light was beneficial for the rooting of *C. lanceolata* tissue culture seedlings. A ratio of 8:1:1:1 was the most ideal composition.

The composite LED light is beneficial for the accumulation of SSC in the leaves of *C. lanceolata* tissue culture seedlings, which relates to the regulation of sucrose-metabolizing enzymes by photo-induced phytochrome^42^. Under the red-blue composite light, the SPC and FPC with 4R1B treatment were significantly higher than those with the 8R1B treatment. Blue light can inhibit the activity of α-ketoglutarate dehydrogenase, activate nitrate reductase and promote the synthesis of pyruvate kinase protein within the glycolysis pathway. Meanwhile, the photon energy in the blue light spectrum is higher, which can provide more energy for the synthesis of macromolecules such as proteins^43–45^. Therefore, the larger the proportion of blue light within red-blue composite light is, the greater the SPC and FPC in plant leaves is. This is also true for tobacco plants^46^ and Chinese chive plants^47^. However, the SPC and FPC under the 6R1B1P1G treatment was significantly lower compared to 8R1B1P1G treatment, indicating that the addition of purple and green lights on the basis of red-blue composite light shifted the effect of red-blue composite light. Moreover, the SPC under the 8R1B1P, 6R1B1P1G, and 8R1B1P1G treatments was higher compared to 4R1B and 8R1B treatments, indicating that purple and green lights can synergize red and blue lights and result in an increased SPC. Thus, the regulation of light quality on the SPC and FPC concentration in tissue culture seedlings is a complex process, without strict positive or negative correlations between effect and light quality^48^.

The oxidizing substances in plants are predominantly derived from photosynthesis within chloroplasts, and respiration in mitochondria. Insufficient utilization of light energy during photosynthesis results in accumulation of oxidative active substances in plants^49^. The MDA is one of the products of cell membrane peroxidation, which reflects the antioxidant capacity of plants^50^. Antioxidant enzymes play an important role in the removal of reactive oxygen in plants, and the activity reflects the physiological activity and senescence of plants^51,52^. The SOD is the first line of defense against oxidative stress and lipid peroxidation, which catalyzes the formation of H_2_O_2_ and O_2_ from O_2_^−^; the POD and CAT can remove H_2_O_2_ from peroxisomes and cytoplasm^53,54^. In this study, the MDA content was lower in the 6R1B1P1G and 8R1B1P1G treatment groups, while the activities of SOD, POD and CAT were higher, indicating that the combination of red-blue-purple-green composite light could effectively improve the antioxidant capacity of tissue culture seedlings and delay plant senescence. The APX can catalyze the oxidation of ascorbic acid and scavenge reactive oxygen species through the ascorbate-glutathione-NADPH cycle pathway. The APX activity after 8R1B and 8R1B1P1G treatment was significantly higher compared to 4R1B and 6R1B1P1G treatments, indicating that the composite light with a higher red light ratio was more favorable for the increase of APX activity. This may be due to the fact that APX is more sensitive to red light^16^. PPO can catalyze the oxidation of polyphenols to strontium in plants, which polymerizes and reacts with amino acids in intracellular proteins, resulting in melanin precipitation and plant browning. Our results show that the PPO activity under 6R1B1P1G and 8R1B1P1G treatments was lower, suggesting that the culture seedlings were less likely to brown under this composite light, ultimately leading to healthier plants.

Light is an important signal element that regulates plant morphology, physiology and development in plant growth cycle. Light signals are perceived by complex plant photoreceptor systems. These photoreceptors mainly exist in leaves. The system coordinates with proteins, ions, hormones and other factors to regulate gene expression patterns, metabolic reactions and accompanying changes in plant morphology^20,36^. Gupta and Agarwal^36^ pointed out that the regulation of reactive oxygen species (ROS) and their interaction with antioxidant system are one of the important mechanisms affecting plant growth and morphogenesis. In this study, the root growth parameters and antioxidant capacity parameters of tissue culture seedlings to different light qualities were different. And the root growth parameters of tissue culture seedlings was closely related to the antioxidant capacity parameters. The POD activity positively correlated with root activity; the SPC, the SOD and CAT activities positively correlated with root growth parameters, while the SSC and MDA, and APX and PPO activities negatively correlated to root growth parameters. Root growth was the best under 8R1B1P1G light treatment, and the activity of antioxidant enzymes was higher. This may be due to the fact that the light quality creates a high oxidative stress environment for *C. lanceolata* leaves, and the synergistic effects of SSC, SPC, FPC metabolism and antioxidant enzymes system make the plant environment in a relatively stable state, promote the photosynthesis of leaves and the distribution of photosynthates to roots, thus promoting root growth. This study preliminarily determined the effects of composite LED light quality and its ratio on the root growth and antioxidant capacity of the *C. lanceolata* tissue culture seedlings. Light quality regulates the synthesis of certain functional proteins or enzymes by regulating its gene expression, which changes the physiological metabolism of tissue culture seedlings that resulted in altered root growth and development^55,56^. Therefore, future research is aimed at in-depth understanding of the mechanism of gene regulation and protein expressions on the basis of mastering basic laws.

## Materials and Methods

### Experiment design

The experiment was conducted at the Forestry college of Guangxi university, China between March and June 2016. The 12^th^ generation of *C. lanceolata* tissue culture seedlings were used as study material, and within the growing seedlings, plants with a height of approximately 1.5 cm were selected. The seedlings were pre-cultured in the growth medium (Murashige and Skoog medium (MS) + Indolebutyric acid (IBA) 0.3 mg/L + 6-benzylaminopurine (6-BA) 0.6 mg/L + Kinetin (KT) 0.1 mg/L + Sucrose 30 g/L + Agar 5.5 g/L + Activated carbon 0.3 g/L) for 10 days, and were then transplanted to the rooting medium (1/4 MS + IBA 0.7 mg/L+ Naphthylacetic acid (NAA) 0.2 mg/L + ABT root-inducing regulator (ABT1^#^) 0.2 mg/L +Sucrose 15.0 g/L + Agar 5.5 g/L). The pH value of both media was 6.0. The LED light peaks for red are 620–630 nm, blue 460–470 nm, purple 410–420 nm, and green 520–530 nm. Five composite LED light treatments, 4R1B, 8R1B, 8R1B1P, 6R1B1P1G, and 8R1B1P1G, were used for our study, with white LED light used as control (CK). All of the above light sources were provided by Shenzhen Weixinli Photoelectric Co., Ltd. China. Each group contained two corresponding lamps and different chambers were separated with a blackout cloth. The current and distance between the light source and the plant was adjusted so that the light intensity of the upper surface of the bottle seedlings was consistent (approximately 600–700 lx) in all treatment groups. 15 bottles of rooting stage tissue culture seedlings (3 plants per bottle) were used per treatment, with 3 replicates per group, and cultured for 30 d. The photoperiod was 16/8 h (day/night), day/night temperature was 25 °C ± 1 °C, and relative humidity was 60 ± 5%.

The growth medium, rooting medium and the distance between light source and plant used in this experiment all came from previous experiments (Unpublished data, see Supplementary Information).

### Root growth parameters determination

The total number of seedlings, rooting seedlings and roots per treatment were counted, and the rooting rate and average number of roots were calculated accordingly. Epson Expression^TM^ 10000 XL (Seiko Epson Corporation, Nagano, Japan) was used to determine the length, surface area, and volume of roots and 10 seedlings were randomly selected for each treatment group. Data was analyzed using Win RHIZOC Pro 2004 b software. The calculation formula is as follows:

$$\begin{array}{c}{\rm{rooting}}\,{\rm{rate}}={\rm{number}}\,{\rm{of}}\,{\rm{rooting}}\,{\rm{seedlings}}/{\rm{total}}\,{\rm{number}}\,{\rm{of}}\,{\rm{seedlings}}\times 100 \% ;\\ {\rm{average}}\,{\rm{number}}\,{\rm{of}}\,{\rm{roots}}={\rm{total}}\,{\rm{number}}\,{\rm{of}}\,{\rm{roots}}/{\rm{total}}\,{\rm{number}}\,{\rm{of}}\,{\rm{seedlings}}.\end{array}$$

### Antioxidant determination

One gram of fresh roots were randomly selected from each treated seedling, and the root activity was determined using the Triphenyl tetrazolium chloride method (TTC) method^57^. One gram of fresh leaves were randomly selected from each treated seedling to determine antioxidant capacity parameters, and 3 replicates per treatment group were analyzed. According to Chen and Wang^58^, the soluble sugar content (SSC) was determined using the indolinone sulfuric acid method; the free proline content (FPC) was determined by the acid ninhydrin method; the soluble protein content (SPC) was determined by spectrophotometry; the malondialdehyde (MDA) content was determined using thiobarbituric acid and the superoxide dismutase (SOD) activity was determined with nitrogen blue tetrazolium (NBT). An inhibition of nitroblue tetrazolium by 50% was defined as 1 enzyme activity unit, expressed as terms of fresh mass enzyme units per gram (U/g, 1 U = 16.67 nkat). The peroxidase (POD), catalase (CAT), peroxidase (APX) and polyphenol oxidase (PPO) were measured using UV spectrometry^58^. Regarding the enzyme activity, the amount of enzyme required to change 0.01 absorbance values in 1 min was defined as 1 active unit, represented in as U/(min·g) (U = 16.67 nkat). Each index was determined using 3 replicates per treatment groups.

### Data statistics and analysis

Data analysis was performed using SPSS 21.0 software (IBM Inc., Chicago, IL, USA), including One-Way ANOVA and Duncan multiple comparisons. The Origin 2017 SR2 software (Origin Lab Inc., Hampton, MA, USA) was used for plotting.

In this study, redundancy analysis (RDA) was used to explore the relationship between root growth parameters and antioxidant capacity parameters. The data was subject to correspondence analysis (DCA), and the maximum value of the four sorting axis gradient lengths (LGA) was 1.31 (<3), indicating that these data was suitable for redundant analysis using a linear model. The above analyses carried out using Canoco 5.0 software.

## Supplementary information

Related Manuscript File
