# Supplementary material for: Effects of Composite LED Light on Root Growth and Antioxidant Capacity of Cunninghamia lanceolata Tissue Culture Seedlings
Source: Sci Rep. 2019 Jul 5;9:9766. doi: 10.1038/s41598-019-46139-2 (PMC6611763; doi:10.1038/s41598-019-46139-2)
Supplement: Supplementary file 1 — Related Manuscript File [file 41598_2019_46139_MOESM1_ESM.docx]

**Effects of Composite LED Light on Root Growth and Antioxidant Capacity of *Cunninghamia lanceolata* Tissue Culture Seedlings**

Yuanyuan Xu ^1, 2^, Yuyao Liang ^3^, Mei Yang ^1, *^

^1^ Guangxi Key Laboratory of Forest Ecology and Conservation, College of Forestry, Guangxi University, Nanning, 530004, Guangxi, PR China. ^2^ College of Forestry, Beijing Forestry University, Beijing, 100083, PR China. ^3^College of resources and environment, Fujian Agriculture and Forestry University, Fuzhou, 350002, Fujian, PR China.

(^*^corresponding. email: fjyangmei@126.com)

**Supplementary information**

The previous experiments were carried out as the reference of the experiment designs of the research in our paper. To explore the effects of different plant growth regulators, LED photoperiod and light quality on the rooting of *C. lanceolata* tissue culture seedlings, the following experimental were carried out according to the research:

**1. Rooting of tissue culture seedlings under different light intensities**

**Objective:** to screen suitable light intensity for rooting culture of tissue cultured seedlings of *C. lanceolate*.

**Study materials:** *C. lanceolata* tissue culture seedlings (height: 1.5 cm).

**LED light quality:** red-blue 4:1 (recorded as 4R1B).

**LED photoperiod:** 12 h/d (day/night).

**Light intensity:** 600-700 lx, 1000-1200 lx.

**Temperature:** 25±1 ℃.

**Relative humidity:** 60±5%.

Cultured for 30 d.

**Growth medium:** 1/4 Murashige and Skoog medium (MS)+ Indolebutyric acid (IBA) 0.9 mg/L+ Naphthylacetic acid (NAA) 0.4 mg/L+ Sucrose 20 g+ Agar 5.5 g, pH 6.0.

**Conclusion:** The optimum light intensity is 600-700 lx (Table 1).

Table 1. Rooting of tissue culture seedlings under different light intensities

| Light intensity/lx | Rooting rate/% | Average root | Root length/cm |
| --- | --- | --- | --- |
| 600-700 | 70.52±1.35 | 2.51±0.52 | 1.92±0.46 |
| 1000-1200 | 56.60±1.63 | 1.89±0.44 | 1.25±0.54 |

**2. Rooting of tissue culture seedlings under different rooting medium**

**Objective:** to screen suitable rooting medium for rooting culture of tissue cultured seedlings of *C. lanceolate*.

**Study materials:** *C. lanceolata* tissue culture seedlings (height: 1.5 cm).

**LED light quality:** red-blue 4:1, 8:1; White (recorded as 4R1B, 8R1B, W).

**LED photoperiod:** 24 h/d (day/night).

**Light intensity:** 600-700 lx.

**Temperature:** 25±1 ℃.

**Relative humidity:**60±5%.

Cultured for 30 d.

**Rooting medium:**

①1/4 MS+IBA 1.0 mg/L+ ABT root-inducing regulator (ABT1^#^) 1.0 mg/L+ Sucrose 20 g/L + Agar 5.5 g/L

②1/4 MS+IBA 0.9 mg/L+NAA 0.4 mg/L+ Sucrose 20 g/L + Agar 5.5 g/L

③1/4 MS+IBA 1.0 mg/L+ Sucrose 20 g/L + Agar 5.5 g/L

④1/4 MS+NAA 1.0 mg/L+ Sucrose 20 g/L + Agar 5.5 g/L

⑤1/4 MS+IBA 0.7 mg/L+NAA 0.2 mg/L+ABT 1^#^ 0.1 mg/L+ Sucrose 20 g/L + Agar 5.5 g/L

**Conclusion:** Tissue culture seedlings had higher rooting rate with growth medium ②, ⑤ than other treatments (Table 2).

**3. Rooting of tissue culture seedlings under different rooting medium and different light quality**

**Objective:** to screen suitable rooting medium and light quality for rooting culture of tissue-cultured seedlings of *C. lanceolate*.

**Study materials:** *C. lanceolata* tissue culture seedlings (height: 1.5 cm).

**LED light quality:** red-blue 2:1, 4:1, 6:1, 8:1, 10:1 (recorded as 2R1B, 4R1B, 6R1B, 8R1B, 10R1B); red-blue-purple 4:1:1, 6:1:1, 8:1:1 (recorded as 4R1B1P, 6R1B1P, 8R1B1P); red-blue-purple-green 6:1:1:1, 8:1:1:1 (recorded as 6R1B1P1G, 8R1B1P1G).

**LED photoperiod:** 12 h/d (day/night).

**Light intensity:** 600-700 lx.

**Temperature:** 25±1 ℃.

**Relative humidity:** 60±5%.

Cultured for 30 d.

**Rooting medium:**

①1/4 MS+IBA 1.1 mg/L+NAA 0.6 mg/L+ Sucrose 15 g/L + Agar 5.5 g/L

②1/4 MS+IBA 0.9 mg/L+NAA 0.4 mg/L+ Sucrose 15 g/L + Agar 5.5 g/L

③1/4 MS+IBA 0.7 mg/L+NAA 0.2 mg/L + Sucrose 15 g/L + Agar 5.5 g/L

④1/4 MS+IBA 0.7 mg/L+NAA 0.2 mg/L+ABT 1^#^ 0.2 mg/L+ Sucrose 15 g/L + Agar 5.5 g/L

**Conclusion:** Overall, the root growth of tissue culture seedlings under growth medium ④ was better than other treatments. At the same time, LED light quality 4R1B, 8R1B, 8R1B1P, 6R1B1P1G, 8R1B1P1G have higher research value (Table 3).

**To sum up, we choose the following conditions for our research, including:**

**Study materials:** *C. lanceolata* tissue culture seedlings (height: 1.5 cm).

**LED light quality:** red-blue 4:1, 8:1 (recorded as 4R1B, 8R1B); red-blue-purple 8:1:1 (recorded as 8R1B1P); red-blue-purple-green 6:1:1:1, 8:1:1:1 (recorded as 6R1B1P1G, 8R1B1P1G).

**LED photoperiod:** 12 h/d (day/night).

**Light intensity:** 600-700 lx.

**Temperature:** 25±1 ℃.

**Relative humidity:** 60±5%.

Cultured for 30 d.

**Rooting medium:** 1/4 MS+IBA 0.7 mg/L+NAA 0.2 mg/L+ABT 1^#^ 0.2 mg/L+ Sucrose 15 g/L + Agar 5.5 g/L

Table 2. Rooting of tissue culture seedlings under different rooting medium

| Light quality | ① | | | ② | | | ③ | | | ④ | | | ⑤ | | |
| --- | --- | --- | --- | --- | --- | --- | --- | --- | --- | --- | --- | --- | --- | --- | --- |
|  | Rooting rate/% | Average root | Root length/cm | Rooting rate/% | Average root | Root length/cm | Rooting rate/% | Average root | Root length/cm | Rooting rate/% | Average root | Root length/cm | Rooting rate/% | Average root | Root length/cm |
| 4R1B | 33.7±  1.15 b | 1.10±  0.10 c | 1.31±0.06 a | 72.67±1.17 b | 2.39±  0.07 a | 1.82±0.09 a | 42.57±0.85 c | 1.91±  0.07 a | 1.17±  0.31 a | 9.23±  0.41 c | 0.89±  0.03 c | 1.27±  0.13 a | 87.67±1.47 a | 0.89±  0.03 b | 1.03±  0.16 ab |
| 8R1B | 64.27±  1.89 a | 1.31±  0.05 b | 1.28±0.04 a | 90.83±2.02 a | 1.21±  0.04 c | 1.52±0.10 b | 54.23±0.49 b | 1.61±  0.11 b | 1.39±  0.03 a | 45.67±1.47 b | 0.91±  0.07 b | 1.23±  0.06 a | 76.57±0.67 b | 1.22±  0.03 a | 1.23±  0.03 a |
| W | 36.60±  1.78 b | 1.51±  0.04 a | 1.29±0.04 a | 50.93±1.01 c | 1.31±  0.04 b | 1.13±0.11 c | 56.43±1.32 a | 1.31±  0.06 c | 1.54±  0.07 a | 60.60±1.04 a | 1.17±  0.12 a | 0.94±  0.07 b | 61.93±1.67 c | 1.10±  0.11 a | 0.91±  0.07 b |

Note: mean ± *SD*; Different small letters in the same column indicate significant differences among light quality (*P*＜0.05, Duncan’s multiple range test).

Table 3 Rooting of tissue culture seedlings under different rooting medium and different light quality

| Light quality | ① | | | ② | | | ③ | | | ④ | | |
| --- | --- | --- | --- | --- | --- | --- | --- | --- | --- | --- | --- | --- |
|  | Rooting rate/% | Average root | Root length/cm | Rooting rate/% | Average root | Root length/cm | Rooting rate/% | Average root | Root length/cm | Rooting rate/% | Average root | Root length/cm |
| 2R1B | 3.61±  0.20 fg | 0.11±  0.02 bc | 0.21±  0.02 d | 20.11±  0.20 g | 0.35±  0.08 g | 0.41±  0.02 f | 71.51±  1.93 b | 2.01±  0.20 a | 0.55±  0.08 d | 44.45±  0.77 e | 1.31±  0.20 c | 0.44±  0.08 d |
| 4R1B | 4.53±  0.23 f | 0.11±  0.01 bc | 0.43±  0.05 d | 37.40±  1.21 d | 0.48±  0.03 f | 0.33±  0.05 g | 81.07±  0.75 a | 1.89±  0.02 a | 0.51±  0.02 de | 30.93±  0.64 f | 0.89±  0.04 e | 0.48±  0.03 d |
| 6R1B | 11.53±  0.46 d | 0.11±  0.01 bc | 0.87±  0.06 b | 26.87±  0.75 f | 0.58±  0.03 f | 0.48±  0.03 f | 58.87±  0.75 d | 1.54±  0.10 b | 0.49±  0.02 de | 6.96±  5.27 h | 0.21±  0.02 g | 0.11±  0.01 f |
| 8R1B | 3.33±  0.29 g | 0.11±  0.02 bc | 0.29±  0.02 de | 16.13±  0.98 h | 0.17±  0.05 h | 1.47±  0.05 a | 51.33±  0.98 fg | 1.62±  0.03 b | 0.48±  0.03 de | 27.40±  1.04 g | 0.56±  0.07 f | 0.68±  0.03 c |
| 10R1B | 12.07±  0.75 d | 0.09±  0.01 c | 0.38±  0.03 d | 32.87±  0.75 e | 0.77±  0.05 e | 0.49±  0.02 f | 59.40±  1.04 g | 1.13±  0.06 cd | 0.55±  0.09 d | 27.73±  1.50 fg | 0.57±  0.06 f | 0.49±  0.01 d |
| 4R1B1P | 3.07±  0.05 g | 0.11±  0.01 bc | 0.42±  0.03 d | 14.87±  0.58 h | 0.21±  0.01 h | 0.19±  0.01 h | 16.27±  0.75 h | 0.29±  0.01 f | 0.48±  0.04 de | 24.73±  0.46 g | 0.38±  0.03 fg | 0.19±  0.02 e |
| 6R1B1P | 9.60±  0.69 e | 0.19±  0.01 a | 0.37±  0.06 de | 60.73±  0.64 c | 1.07±  0.05 d | 0.76±  0.07 e | 53.73±  1.50 e | 1.07±  0.06 d | 0.67±  0.06 c | 51.47±  1.27 d | 0.79±  0.01 e | 0.82±  0.04 b |
| 8R1B1P | 3.88±  0.02 fg | 0.10±  0.01 bc | 0.59±  0.02 c | 75.27±  2.83 b | 1.46±  0.07 c | 0.69±  0.02 e | 49.40±  1.04 g | 0.87±  0.05 e | 0.58±  0.03 cd | 58.73±  0.64 c | 1.27±  0.06 cd | 1.19±  0.02 a |
| 6R1B1P1G | 61.10±  0.82 a | 0.11±  0.01 bc | 1.13±  0.23 a | 73.53±  1.32 b | 1.86±  0.07 b | 0.87±  0.06 d | 72.67±  1.44 b | 1.89±  0.18 a | 0.86±  0.07 b | 90.93±  1.67 a | 3.01±  0.15 a | 0.88±  0.03 b |
| 8R1B1P1G | 47.73±  0.23 b | 0.20±  0.02 a | 1.12±  0.11 a | 76.37+  1.23 a | 2.09±  0.09 a | 1.16±  0.07 b | 68.33±  1.33 c | 1.53±  0.06 b | 1.09±  0.10 a | 83.83±  1.32 b | 2.05±  0.27 b | 1.14±  0.07 a |
| W | 33.30±  1.30 c | 0.13±  0.04 b | 0.77±  0.12 b | 38.93±  0.81 d | 1.07±  0.12 d | 1.03±  0.05 c | 52.87±  0.75 ef | 1.29±  0.02 c | 0.42±  0.03 e | 59.40±  1.22 c | 1.09±  0.01 d | 0.87±  0.06 b |

Note: mean ± *SD*; Different small letters in the same column indicate significant differences among light quality (*P*＜0.05, Duncan’s multiple range test).
